# 1-Methyl-1-propyl­pyrrolidinium chloride

**DOI:** 10.1107/S1600536808005229

**Published:** 2008-02-29

**Authors:** Pamela M. Dean, Jennifer M. Pringle, Douglas R. MacFarlane

**Affiliations:** a School of Chemistry, Monash University, Wellington Road, Clayton, Victoria 3800, Australia

## Abstract

The aymmetric unit of the title compound, C_8_H_18_N^+^·Cl^−^, consists of one crystallographically independent 1-methyl-1-propyl­pyrrolidinium cation and one chloride anion, both of which lie in general positions. Minor hydrogen-bonded C—H⋯Cl inter­actions occur. However, no classical hydrogen bonding is observed.

## Related literature

For bond-length data, see: Allen *et al.* (1987 ▶). For comparative thermal and crystallographic analysis of four crystallized *N*-alkyl-*N*-methyl­pyrrolidinium and piperidinium bis­(trifluoro­methane­sulfon­yl)imide salts and an insight into why these salts form room-temperature ionic liquids, see: Henderson *et al.* (2006 ▶). For the synthesis and analysis of *N*-butyl-*N*-methyl pyrrolidinium chloride, an analogue of the title compound, see: Lancaster *et al.* (2002 ▶). For the first synthesis and analysis of the new pyrrolidinium family of molten salts, see: MacFarlane *et al.* (1999 ▶). For the quanti­tative comparison of inter­molecular inter­actions using Hirshfeld surfaces, see: McKinnon *et al.* (2007 ▶). For the first synthesis and analysis of 1-alkyl-2-methyl pyrrolidinium ionic liquids involving the bis­(trifluoro­methane­sulfon­yl)imide anion, see: Sun *et al.* (2003 ▶).
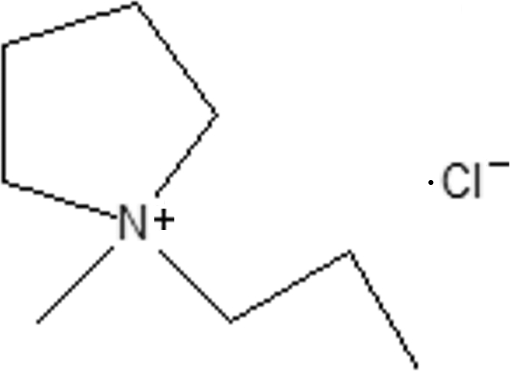


## Experimental

### 

#### Crystal data

C_8_H_18_N^+^·Cl^−^
*M* *_r_* = 163.68
Orthorhombic, 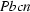

*a* = 14.5863 (5) Å
*b* = 13.2196 (4) Å
*c* = 9.9779 (3) Å
*V* = 1923.99 (11) Å^3^
*Z* = 8
Mo *K*α radiation
μ = 0.33 mm^−1^
*T* = 123 (2) K
0.30 × 0.30 × 0.30 mm

#### Data collection

Bruker Kappa APEXII diffractometer
Absorption correction: multi-scan (*SADABS*; Bruker, 2005 ▶) *T*_min_ = 0.907, *T*_max_ = 0.907
11550 measured reflections
1982 independent reflections
1800 reflections with *I* > 2σ(*I*)
*R*_int_ = 0.030

#### Refinement

*R*[*F* ^2^ > 2σ(*F* ^2^)] = 0.031
*wR*(*F* ^2^) = 0.079
*S* = 1.04
1982 reflections
93 parameters
H-atom parameters constrained
Δρ_max_ = 0.25 e Å^−3^
Δρ_min_ = −0.20 e Å^−3^

### 

Data collection: *APEX2* (Bruker, 2005 ▶); cell refinement: *APEX2*; data reduction: *APEX2*; program(s) used to solve structure: *SHELXS97* (Sheldrick, 2008 ▶); program(s) used to refine structure: *SHELXL97* (Sheldrick, 2008 ▶); molecular graphics: *POV-RAY* (Persistence of Vision, 2003 ▶); software used to prepare material for publication: *SHELXL97*.

## Supplementary Material

Crystal structure: contains datablocks I, global. DOI: 10.1107/S1600536808005229/zl2101sup1.cif

Structure factors: contains datablocks I. DOI: 10.1107/S1600536808005229/zl2101Isup2.hkl

Additional supplementary materials:  crystallographic information; 3D view; checkCIF report

## Figures and Tables

**Table 1:** Hydrogen-bond geometry (Å, °)

*D*—H⋯*A*	*D*—H	H⋯*A*	*D*⋯*A*	*D*—H⋯*A*
C1—H1*B*⋯Cl1^i^	0.99	2.79	3.607 (1)	141
C2—H2*A*⋯Cl1^ii^	0.99	2.77	3.630 (2)	146
C5—H5*A*⋯Cl1	0.98	2.77	3.648 (1)	149
C5—H5*C*⋯Cl1^iii^	0.98	2.71	3.656 (1)	163
C6—H6*A*⋯Cl1^i^	0.99	2.76	3.672 (1)	153
C6—H6*B*⋯Cl1	0.99	2.77	3.666 (1)	151

Symmetry codes: (i) 
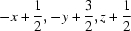
; (ii) 
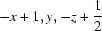
; (iii) 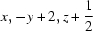.

**Fig. 1.**
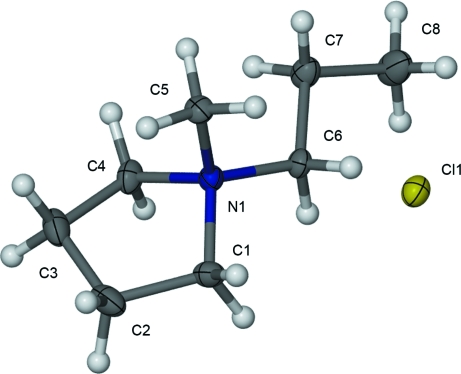
Diagram of the unique component of (I) shown with 50% thermal ellipsoids and hydrogen atoms as spheres of arbitrary size.

**Fig. 2.**
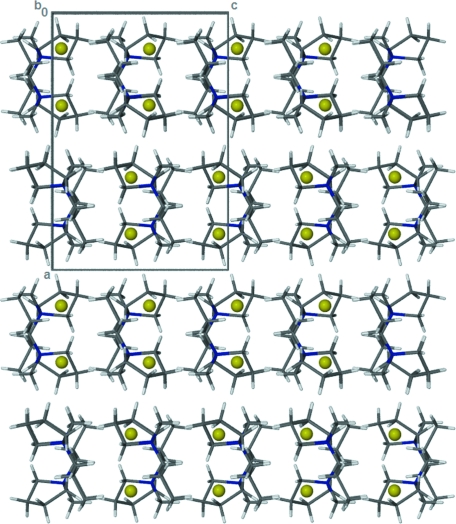
Extended packing diagram of the unit-cell contents of (I) as viewed down the b axis.

## supplementary crystallographic information

### Comment

The title compound, (I), is commonly used as a precursor in ionic liquid synthesis (MacFarlane *et al.*, 1999, Sun *et al.*, 2003). Pyrrolidinium-based ionic liquids have been a subject of intense investigation recently (Henderson *et al.*, 2006), whereby with understanding of the fundamental molecular-level interactions, a desired product with predicted physico-chemical properites could be designed. Additionally, a particular emphasis has been placed on whether hydrogen bonding occurs between the cation and a potential electron-pair donor (hydrogen bond acceptor) and its influence on the ionic liquids' overall properties. This paper briefly reports the structural determination and analysis of 1-methyl-1-propyl pyrrolidinium chloride (Figure 1).

The bond distances and angles of the pyrrolidinium cation are all within normal ranges (as is tabulated in Allen *et al.*, 1987), with the propyl substituent adopting the energetically preferred *anti* conformation (torsional angle N1—C6—C7—C8: -177.0 (2) °) and the ring adopting the energetically preferred envelope (C*s*) conformation. The extended structure packs in layers of groups of anions and cations (Figure 2) which are interconnected by an extended network of weak hydrogen bonds (C—H···Cl interactions), where each cation is hydrogen bonded to four anions and each anion is weakly hydrogen bonded to four cations [C1—H1B···Cl1^i^ [3.607 (2) Å], C2—H2A···Cl1^ii^ [3.630 (2) Å], C5— H5A ··· Cl1 [3.648 (1) Å], C5—H5C···Cl1^iii^ [3.656 (1) Å], C6—H6A···Cl1^i^ [3.672 (2) Å] and C6— H6B···Cl1 [3.666 (1) Å] (symmetry operators: i=1/2 - *x*,3/2 - *y*,1/2 + *z*; ii=1 - *x*,*y*,1/2 - *z*; iii=*x*,2 - *y*,1/2 + *z*) -see Table 1]. Analysis of the salts' Hirshfeld surface, reveals that the short range inter-cationic H—H intermolecular contact contribution to the Hirshfeld surface area predominates (McKinnon *et al.*, 2007).

### Experimental

The compound was synthesized following the procedure of Lancaster *et al.* (2002) for the analogous *N*-butyl-*N*-methyl pyrrolidinium chloride species: 1-methyl-1-propylpyrrolidinium chloride was synthesized by heating a solution of chloropropane (28 ml, 0.315 moles) and methyl pyrrolidine (20 ml, 0.287 moles) in 2-propanol at 323 K under nitrogen for 48 h. The resultant white solid was recrystallized from 2-propanol at 273 K. Crystals resulted after 2 days. Crystals were coated with Paratone N oil (Exxon Chemical Co., TX, USA) immediately after isolation and cooled in a stream of nitrogen vapour on the diffractometer. Melting point: 323.5 K.

### Refinement

All H atoms were initially located in a difference Fourier map. Thereafter, all H atoms were placed in geometrically fixed idealized positions and constrained to ride on their parent atoms with C—H distances in the range 0.95–1.00 Å and *U*_iso_(H) = 1.2*U*_eq_(C).

### Crystal data

**Table:**

C_8_H_18_N^+^·Cl^–^	*D*_x_ = 1.130 Mg m^−^^3^
*M**_r_* = 163.68	Melting point: 323.5 K
Orthorhombic, *P**b**c**n*	Mo *K*α radiation λ = 0.71073 Å
Hall symbol: -P 2n 2ab	Cell parameters from 4825 reflections
*a* = 14.5863 (5) Å	θ = 2.8–26.4º
*b* = 13.2196 (4) Å	µ = 0.33 mm^−^^1^
*c* = 9.9779 (3) Å	*T* = 123 (2) K
*V* = 1923.99 (11) Å^3^	Cubic, colourless
*Z* = 8	0.30 × 0.30 × 0.30 mm
*F*_000_ = 720	

### Data collection

**Table:**

Bruker X8 APEX KappaCCD diffractometer	1982 independent reflections
Radiation source: fine-focus sealed tube	1800 reflections with *I* > 2σ(*I*)
Monochromator: graphite	*R*_int_ = 0.030
*T* = 123(2) K	θ_max_ = 26.4º
0.5° frames in φ and ω scans	θ_min_ = 2.1º
Absorption correction: multi-scan(SADABS; Bruker, 2005)	*h* = −18→18
*T*_min_ = 0.907, *T*_max_ = 0.907	*k* = −15→16
11550 measured reflections	*l* = −11→12

### Refinement

**Table:**

Refinement on *F*^2^	Secondary atom site location: difference Fourier map
Least-squares matrix: full	Hydrogen site location: inferred from neighbouring sites
*R*[*F*^2^ > 2σ(*F*^2^)] = 0.031	H-atom parameters constrained
*wR*(*F*^2^) = 0.079	*w* = 1/[σ^2^(*F*_o_^2^) + (0.0361*P*)^2^ + 0.834*P*] where *P* = (*F*_o_^2^ + 2*F*_c_^2^)/3
*S* = 1.04	(Δ/σ)_max_ = 0.001
1982 reflections	Δρ_max_ = 0.25 e Å^−^^3^
93 parameters	Δρ_min_ = −0.20 e Å^−^^3^
Primary atom site location: structure-invariant direct methods	Extinction correction: none

### Special details

**Table:**

Geometry. All e.s.d.'s (except the e.s.d. in the dihedral angle between two l.s. planes) are estimated using the full covariance matrix. The cell e.s.d.'s are taken into account individually in the estimation of e.s.d.'s in distances, angles and torsion angles; correlations between e.s.d.'s in cell parameters are only used when they are defined by crystal symmetry. An approximate (isotropic) treatment of cell e.s.d.'s is used for estimating e.s.d.'s involving l.s. planes.
Refinement. Refinement of *F*^2^ against ALL reflections. The weighted *R*-factor *wR* and goodness of fit *S* are based on *F*^2^, conventional *R*-factors *R* are based on *F*, with *F* set to zero for negative *F*^2^. The threshold expression of *F*^2^ > σ(*F*^2^) is used only for calculating *R*-factors(gt) *etc*. and is not relevant to the choice of reflections for refinement. *R*-factors based on *F*^2^ are statistically about twice as large as those based on *F*, and *R*- factors based on ALL data will be even larger.

### Fractional atomic coordinates and isotropic or equivalent isotropic displacement parameters (Å^2^)

**Table:**

	*x*	*y*	*z*	*U*_iso_*/*U*_eq_	
Cl1	0.36024 (2)	0.82436 (2)	0.05218 (3)	0.02423 (12)	
N1	0.32912 (7)	0.86923 (8)	0.43497 (10)	0.0179 (2)	
C1	0.35707 (9)	0.75928 (10)	0.41992 (14)	0.0241 (3)	
H1A	0.3939	0.7496	0.3376	0.029*	
H1B	0.3023	0.7152	0.4152	0.029*	
C2	0.41390 (11)	0.73488 (12)	0.54424 (15)	0.0323 (3)	
H2A	0.4793	0.7268	0.5202	0.039*	
H2B	0.3922	0.6714	0.5863	0.039*	
C3	0.40131 (10)	0.82444 (12)	0.64053 (15)	0.0309 (3)	
H3A	0.3884	0.8004	0.7326	0.037*	
H3B	0.4569	0.8674	0.6423	0.037*	
C4	0.32014 (9)	0.88251 (11)	0.58455 (13)	0.0242 (3)	
H4A	0.2616	0.8540	0.6174	0.029*	
H4B	0.3234	0.9549	0.6097	0.029*	
C5	0.40372 (8)	0.93610 (10)	0.38042 (13)	0.0209 (3)	
H5A	0.4045	0.9315	0.2824	0.031*	
H5B	0.4631	0.9141	0.4161	0.031*	
H5C	0.3922	1.0063	0.4072	0.031*	
C6	0.23958 (8)	0.88722 (10)	0.36358 (13)	0.0198 (3)	
H6A	0.1941	0.8374	0.3962	0.024*	
H6B	0.2487	0.8750	0.2666	0.024*	
C7	0.20062 (9)	0.99281 (11)	0.38223 (14)	0.0254 (3)	
H7A	0.1931	1.0073	0.4789	0.031*	
H7B	0.2433	1.0434	0.3442	0.031*	
C8	0.10858 (10)	0.99984 (12)	0.31221 (18)	0.0372 (4)	
H8A	0.1173	0.9926	0.2153	0.056*	
H8B	0.0806	1.0657	0.3313	0.056*	
H8C	0.0683	0.9458	0.3447	0.056*	

### Atomic displacement parameters (Å^2^)

**Table:**

	*U*^11^	*U*^22^	*U*^33^	*U*^12^	*U*^13^	*U*^23^
Cl1	0.02343 (18)	0.02619 (19)	0.02308 (18)	−0.00399 (13)	−0.00151 (13)	−0.00178 (12)
N1	0.0162 (5)	0.0191 (5)	0.0185 (5)	−0.0015 (4)	−0.0013 (4)	0.0004 (4)
C1	0.0246 (7)	0.0178 (6)	0.0299 (7)	0.0005 (5)	−0.0008 (6)	0.0012 (5)
C2	0.0282 (7)	0.0287 (8)	0.0401 (9)	0.0006 (6)	−0.0051 (6)	0.0123 (6)
C3	0.0273 (7)	0.0406 (9)	0.0247 (7)	−0.0068 (6)	−0.0067 (6)	0.0100 (6)
C4	0.0232 (6)	0.0322 (8)	0.0172 (6)	−0.0044 (6)	−0.0004 (5)	−0.0005 (5)
C5	0.0163 (6)	0.0223 (7)	0.0241 (6)	−0.0031 (5)	0.0008 (5)	0.0023 (5)
C6	0.0157 (6)	0.0231 (6)	0.0206 (6)	−0.0019 (5)	−0.0035 (5)	−0.0014 (5)
C7	0.0208 (6)	0.0257 (7)	0.0298 (7)	0.0018 (5)	−0.0037 (6)	−0.0049 (6)
C8	0.0258 (7)	0.0314 (8)	0.0543 (10)	0.0050 (6)	−0.0138 (7)	−0.0065 (7)

### Geometric parameters (Å, °)

**Table:**

N1—C5	1.5038 (16)	C4—H4B	0.9900
N1—C6	1.5066 (16)	C5—H5A	0.9800
N1—C4	1.5085 (16)	C5—H5B	0.9800
N1—C1	1.5171 (17)	C5—H5C	0.9800
C1—C2	1.526 (2)	C6—C7	1.5185 (18)
C1—H1A	0.9900	C6—H6A	0.9900
C1—H1B	0.9900	C6—H6B	0.9900
C2—C3	1.536 (2)	C7—C8	1.5163 (19)
C2—H2A	0.9900	C7—H7A	0.9900
C2—H2B	0.9900	C7—H7B	0.9900
C3—C4	1.518 (2)	C8—H8A	0.9800
C3—H3A	0.9900	C8—H8B	0.9800
C3—H3B	0.9900	C8—H8C	0.9800
C4—H4A	0.9900		
			
C5—N1—C6	111.31 (10)	N1—C4—H4B	111.0
C5—N1—C4	110.64 (10)	C3—C4—H4B	111.0
C6—N1—C4	111.98 (10)	H4A—C4—H4B	109.0
C5—N1—C1	109.44 (10)	N1—C5—H5A	109.5
C6—N1—C1	109.72 (10)	N1—C5—H5B	109.5
C4—N1—C1	103.46 (10)	H5A—C5—H5B	109.5
N1—C1—C2	105.54 (11)	N1—C5—H5C	109.5
N1—C1—H1A	110.6	H5A—C5—H5C	109.5
C2—C1—H1A	110.6	H5B—C5—H5C	109.5
N1—C1—H1B	110.6	N1—C6—C7	114.30 (10)
C2—C1—H1B	110.6	N1—C6—H6A	108.7
H1A—C1—H1B	108.8	C7—C6—H6A	108.7
C1—C2—C3	106.30 (12)	N1—C6—H6B	108.7
C1—C2—H2A	110.5	C7—C6—H6B	108.7
C3—C2—H2A	110.5	H6A—C6—H6B	107.6
C1—C2—H2B	110.5	C8—C7—C6	109.34 (11)
C3—C2—H2B	110.5	C8—C7—H7A	109.8
H2A—C2—H2B	108.7	C6—C7—H7A	109.8
C4—C3—C2	104.66 (11)	C8—C7—H7B	109.8
C4—C3—H3A	110.8	C6—C7—H7B	109.8
C2—C3—H3A	110.8	H7A—C7—H7B	108.3
C4—C3—H3B	110.8	C7—C8—H8A	109.5
C2—C3—H3B	110.8	C7—C8—H8B	109.5
H3A—C3—H3B	108.9	H8A—C8—H8B	109.5
N1—C4—C3	103.74 (11)	C7—C8—H8C	109.5
N1—C4—H4A	111.0	H8A—C8—H8C	109.5
C3—C4—H4A	111.0	H8B—C8—H8C	109.5
			
C5—N1—C1—C2	85.97 (12)	C1—N1—C4—C3	40.98 (12)
C6—N1—C1—C2	−151.63 (11)	C2—C3—C4—N1	−33.99 (14)
C4—N1—C1—C2	−31.99 (13)	C5—N1—C6—C7	−63.82 (14)
N1—C1—C2—C3	10.96 (15)	C4—N1—C6—C7	60.62 (14)
C1—C2—C3—C4	14.09 (15)	C1—N1—C6—C7	174.90 (11)
C5—N1—C4—C3	−76.14 (13)	N1—C6—C7—C8	−177.02 (12)
C6—N1—C4—C3	159.05 (10)		

### Hydrogen-bond geometry (Å, °)

**Table:**

*D*—H···*A*	*D*—H	H···*A*	*D*···*A*	*D*—H···*A*
C1—H1B···Cl1^i^	0.99	2.79	3.607 (1)	141
C2—H2A···Cl1^ii^	0.99	2.77	3.630 (2)	146
C5—H5A···Cl1	0.98	2.77	3.648 (1)	149
C5—H5C···Cl1^iii^	0.98	2.71	3.656 (1)	163
C6—H6A···Cl1^i^	0.99	2.76	3.672 (1)	153
C6—H6B···Cl1	0.99	2.77	3.666 (1)	151

Symmetry codes: (i) −*x*+1/2, −*y*+3/2, *z*+1/2; (ii) −*x*+1, *y*, −*z*+1/2; (iii) *x*, −*y*+2, *z*+1/2.

## References

[bb1] Allen, F. H., Kennard, O., Watson, D. G., Brammer, L., Orpen, A. G. & Taylor, R. (1987). *J. Chem. Soc. Perkin Trans. 2*, pp. S1–S19.

[bb2] Bruker (2005). *APEX2* Bruker AXS Inc., Madison, Wisconsin, USA.

[bb3] Henderson, W. A., Young, V. G., Pearson, W., Passerini, S., De Long, H. C. & Trulove, P. C. (2006). *J. Phys. Condens. Matter*, **18**, 10377–10390.

[bb4] Lancaster, N. L., Salter, P. A., Welton, T. & Young, G. B. (2002). *J. Org. Chem.* **67**, 8855–8861. 10.1021/jo026113d 12467399

[bb5] MacFarlane, D. R., Meakin, P., Sun, J., Amini, N. & Forsyth, M. (1999). *J. Phys. Chem. B*, **103**, 4164–4170.

[bb6] McKinnon, J. J., Jayatilaka, D. & Spackman, M. A. (2007). *Chem. Commun.* pp. 3814–3816. 10.1039/b704980c 18217656

[bb7] Persistence of Vision (2003). Persistence of Vision Raytracer *POV-RAY* Persistence of Vision Raytracer Pty Ltd, Victoria, Australia. URL: http://www.povray.org/download/.

[bb8] Sheldrick, G. M. (2008). *Acta Cryst.* A**64**, 112–122. 10.1107/S0108767307043930 18156677

[bb10] Sun, J., Forsyth, M. & MacFarlane, D. R. (2003). *Electrochim. Acta*, **48**, 1707–1717.

